# Correlation between symptom experience and fear of cancer recurrence in postoperative breast cancer patients undergoing chemotherapy in China: A cross-sectional study

**DOI:** 10.1371/journal.pone.0308907

**Published:** 2024-09-18

**Authors:** Manxia Han, Huaying Chen, Jialing Li, Xuemei Zheng, Xue Zhang, Lin Tao, Xiaoxia Zhang, Xianqiong Feng

**Affiliations:** 1 Division of Head & Neck Tumor Multimodality Treatment, Cancer Center, West China Hospital, Sichuan University/ West China School of Nursing, Sichuan University, Chengdu, Sichuan Province, China; 2 Cancer Day-care Unit, Division of Medical Oncology, Cancer Center, West China Hospital, Sichuan University/West China School of Nursing, Sichuan University, Chengdu, Sichuan Province, China; 3 Department of Clinical Trial Center, West China Hospital, Sichuan University/ West China School of Nursing, Sichuan University, Chengdu, Sichuan Province, China; 4 Department of Nursing, West China Hospital, Sichuan University/ West China School of Nursing, Sichuan University, Nursing Key Laboratory of Sichuan Province, Chengdu, Sichuan Province, China; University of Technology Sydney, AUSTRALIA

## Abstract

**Objective:**

To analyze the relationship between experience of symptoms (e.g., pain, fatigue) and fear of cancer recurrence (FCR) in Chinese postoperative patients with breast cancer undergoing chemotherapy.

**Methods:**

A total of 225 patients were recruited using convenience sampling. The Fear of Cancer Recurrence Inventory-Chinese Version and the Symptom Experience Index were used to collect data. The Mann–Whitney U test, Spearman’s correlation, and multivariate analysis were employed to explore the relationships between symptom experience and FCR.

**Results:**

The total Fear of Cancer Recurrence Inventory score in postoperative patients with breast cancer undergoing chemotherapy was 43.19±22.83, and >64.0% of participants exhibited significant fear of cancer recurrence. The total score of symptom experience was 27.41±16.77, including scores of symptom severity (16.91±8.70) and symptom distress (10.50±8.89). Participants’ symptom experience was positively correlated with fear of cancer recurrence (*r* = 0.353, P < 0.001). Patients with clinically relevant FCR had higher scores for total symptom experience (Z = -3.911, P<0.001), symptom severity (Z = –3.245, P = 0.001), and symptom distress (Z = –4.185, P<0.001), compared to patients without clinically relevant FCR. Symptom experience (b = 0.511, t = 6.474, P<0.001), age (b = –0.591, t = –4.201, P<0.001), and educational level (b = 4.147, t = 3.955, P<0.001) were statistically correlated with FCR, accounting for 27.0% of the variance. Among these variables, symptom experience demonstrated the strongest correlation, with a beta value of 0.371.

**Conclusion:**

This study followed others in identifying a cross-sectional relationship between symptom experience and FCR. Further prospective research is required to better understand the nature of this relationship.

## Introduction

The number of new cases of breast cancer worldwide reached 2.26 million in 2020, ranking it as the leading malignant tumor, posing the greatest threat to women’s health [1]. During the same period, China recorded 18.4% (410,000) of all new breast cancer cases and 17.1% (117,000) of deaths worldwide [2]. Given the disparities in medical resources, lack of health awareness, changes in reproductive patterns (young people increasingly choosing only one child or nulliparity), and lifestyle changes associated with rapid economic development, cultural, and sociodemographic changes, the age-standardized morbidity and mortality of breast cancer in China are increasing [3]. Conversely, they have decreased in the United States of America, the United Kingdom, and Australia [2, 3]. Breast cancer remains the primary malignant tumor affecting the health of Chinese women.

Fear of cancer recurrence (FCR) is one of the most common unmet cancer needs 5 years after survival [4]. FCR refers to fear and worry related to the possibility of cancer recurrence or progression, and is characterized by five aspects: high levels of concern, worry or intrusive thinking, maladaptation, dysfunction, excessive distress, and difficulty in making plans [5]. One study demonstrated that up to 89% of distressed cancer patients with psychiatric morbidity reported clinically relevant FCR. Importantly, the majority (61%) of patients with clinically relevant FCR lacked psychiatric disorders, suggesting that FCR may be a distinct syndrome, separate from psychological disorders such as anxiety, depression, and adjustment disorders [6]. FCR is common among breast cancer survivors, with an incidence rate of 56–82% in Western populations [7–9]. Despite the high incidence of breast cancer in China, limited research has focused on FCR, with only small-scale studies conducted in economically developed regions. These studies found that approximately 61.0–68.4% of Chinese patients with breast cancer experience clinically significant FCR [10–12]. FCR can persist for several years or even decades [13], and extreme FCR can impair the overall quality of life of breast cancer survivors in long-term (>10 years), leading to reduced treatment compliance, impaired social functioning, increased frequency of hospital visits, and higher medical costs [14, 15]. Further research on FCR in Chinese patients with breast cancer is warranted to address this issue.

Symptom experience refers to the patient’s perception of the frequency, intensity, distress, and meaning of symptoms and emphasizes an individual’s perception of the various dimensions of symptoms [16]. Research has shown that the severity of somatic symptoms (including pain and fatigue) [17], psychological symptoms (depression) [9, 18], and symptom clusters (including psychosomatic, eating-related, or autonomic symptom clusters) [19] are positively correlated with higher levels of FCR. However, it remains unknown whether the symptom experience becomes more severe when certain symptoms worsen or increase in frequency, intensity, distress, or meaning. Therefore, the correlation between the severity of a single symptom or symptom cluster and FCR does not fully reflect the correlation between symptom experience and FCR.

The FCR theory proposed by Simonelli et al. suggests that physical symptoms may act as “cues” or “triggers” signaling disease occurrence or recurrence, which can activate the cognitive responses associated with FCR [20]. Additionally, psychological distress can heighten the awareness of physical symptoms, leading to the misinterpretation of somatic stimuli as signs of recurrence and subsequently inducing FCR [20]. Building on this theoretical model of FCR, we proposed a positive and statistically significant correlation between symptom experience and FCR.

## Materials and methods

### Study design

A cross-sectional survey was conducted at a large general hospital in China.

### Participants

#### Inclusion and exclusion criteria

A total of 240 postoperative patients with breast cancer undergoing chemotherapy at our hospital from January to October 2021 were selected using convenience sampling. Patients diagnosed with breast cancer confirmed by pathology; female patients aged 18 years or older; patients receiving chemotherapy more than once during postoperative treatment; and patients capable of reading and writing were included. Meanwhile, patients with difficulty in verbal communication, unknown disease diagnosis, severe mental illness, tumor recurrence or metastasis, and other severe comorbidities were excluded.

#### Sample size estimation

The formula $n={\left(\frac{z_{\alpha }*\sigma }{\delta }\right)}^{2}$, where δ = 3 (tolerance error), α = 0.05, and σ = 22 (population standard deviation) was used for sample size calculation of quantitative variables in cross-sectional studies, based on the relevant literature and pretest results [21–23]. Considering that 10% of the questionnaires were invalid, the final sample size was 225 participants. A total of 240 questionnaires were distributed, 225 of which were valid. The response rate was 93.7%.

### Research tools

#### Patient general information questionnaire

We designed a questionnaire to collect patient data, including sociodemographic information (age, education level, occupational status, marital status, family income, and personal income) and information related to the disease and treatment (diagnosis time, chemotherapy times, pathological stage, surgical approach, and number of comorbidities).

#### Fear of Cancer Recurrence Inventory-Chinese Version

The Fear of Cancer Recurrence Inventory (FRCI) was developed in 2009 by Simard and Savard in Canada [24]. It consists of 42 items across seven dimensions: triggers (eight items), severity (nine items), psychological distress (four items), coping strategies (nine items), functional impairments (six items), insight (three items), and reassurance (three items). Higher scores indicated a higher level of FCR. The FCRI was adapted into a Chinese version in 2019 by Su Ting [25]. The Cronbach’s α coefficient of the scale was 0.90 for Chinese cancer patients, and the test-retest reliability ranged from 0.71–0.86 [25]. The Cronbach’s α coefficient in this study was 0.953. The severity subscale of the FCRI, known as the Fear of Cancer Recurrence Inventory-Short Form FCRI-SF, is used as a screening tool for FCR because of its strong correlation with the total FCRI score (r = 0.84) [26]. A cutoff score of 13 on the FCRI-SF has been found to distinguish clinically significant FCR, with a specificity of 75% and a sensitivity of 88% [26]. The FCRI-SF has been proven effective in detecting patients with a clinically significant FCR in China [27].

#### Symptom Experience Index

The Symptom Experience Index (SEI) developed by Fu et al. was used to evaluate symptom experience [28]. This index contains 40 items that measure the occurrence or severity of symptoms (20 items) and distress (20 items). The sum of symptom severity and distress scores constituted the total score of symptom experience. Participants were scored on a 5-point Likert-type self-reported scale, with a higher score indicating more severe symptom experiences. The overall Cronbach’s α coefficient was 0.91, and the retest reliability was 0.98 in the Chinese population, including cancer patients [29]. To make the scale more suitable for measuring the symptom experiences of breast cancer patients, we adjusted some of the items and conducted a round of expert consultations. Seven experts, including three clinical nursing experts, three nursing management, three nursing education experts, and one methodological expert, participated in the consultation. The Scale-level Content Validity Index was 0.95, and the item-level content validity Index was 0.98. The revised SEI scale comprised 46 items that assess the occurrence (23 items) and distress (23 items) of symptoms. It did not incorporate many psychological symptoms (e. g., fear, nervousness, low mood) beyond dysphoria, which would offer a more comprehensive and personalized assessment of symptom experiences for breast cancer patients. In this study, it demonstrated Cronbach’s α coefficients of 0.93 and 0.913 in the pre-survey (36 cases) and the formal survey (225 cases), respectively.

#### Data collection

Upon admission, patients received health education in the ward. Thereafter, researchers introduced the study and registered the bed numbers of volunteers. Subsequently, they scheduled a specific time with the participants for the questionnaire survey. To minimize the potential influence of clinical treatment on data collection quality, all surveys were conducted the day before the chemotherapy session, ensuring that no therapeutic or nursing practice occurred on that day. To ensure the quality and response rate of the questionnaire, the researcher personally distributed the printed questionnaires to patients, providing uniform instructions for independent completion. Furthermore, the researcher collected and reviewed the completed questionnaires onsite. Disease-related information was recorded by the investigator by consulting medical records. For patients who faced challenges reading and filling out the questionnaires due to visual issues, the investigator directly communicated with them to complete the questionnaires based on their responses. All questionnaires, reflecting the patients’ experiences and conditions over the preceding month, were collected immediately after completion.

#### Statistical analysis

Data were analyzed using SPSS 25.0 statistical software SPSS 25.0 (IBM Corp., Armonk, NY, USA). Statistical significance was set at a two-sided P < 0.05. Categorical variables are presented as absolute values and percentages. Continuous variables with a normal distribution are reported as median with standard deviation, while those with a skewed distribution are reported as median with 75th and 25th percentile values. Analysis of variance and t-tests were used to explore differences in FCR between the patient characteristics. The chi-square test was used to compare characteristics of patients with (FCRI-SF score ≥13) and without (FCRI-SF <13) clinically significant FCR. Because the data exhibited a skewed distribution, the Mann–Whitney U test was employed to compare the differences in FCR and symptom experience between the groups with and without clinically significant FCR. Spearman’s correlation test was used to explore the correlation between symptom experience and FCR. Multiple linear regression analysis (stepwise method) was conducted to explore the primary factors influencing FCR.

#### Ethical approval

Ethical approval for the study was obtained from the Biomedical Ethics Review Committee of West China Hospital of Sichuan University in 2021 (No.1116). The study adhered to the principles of biomedical ethics, and informed consent was obtained from all participants before the investigation. All informed consent statements are attached to the questionnaire homepage. Participants could decide whether to participate in the study and were free to withdraw at any time without facing punitive measures. All questionnaire materials were securely stored in a locked cabinet accessible only to the investigator. All data will remain confidential to individuals other than the participants after anonymous analysis.

## Results

### Characteristics of participants with or without clinically significant FCR

A total of 240 patients with breast cancer undergoing postoperative chemotherapy participated in the survey. Of these, 225 surveys were used for the data analysis. The differences between demographic characteristics as well as disease and treatment-related information with or without clinically significant FCR are shown in Table 1. Occupational status was the only difference in the basic data between the groups of patients with or without clinically significant FCR.

**Table 1:** Difference of basic participants’ data with or without FCR clinical significance (N = 225).

Variable		FCRI-SF<13	FCRI-SF≧13	*Χ* ^*2*^	*P*
		n (%)	n (%)		
**Age, year**				9.912	0.42
	≤30	1 (0.4)	7 (3.1)		
	31–40	5 (2.2)	23 (10.2)		
	41–50	35 (15.6)	56 (24.9)		
	51–60	25 (11.1)	45 (20.0)		
	>60	15 (6.7)	13 (5.8)		
**Educational level**				11.086	0.26
	Elementary school or below	14 (6.2)	13 (5.8)		
	Junior high school	24 (10.7)	51 (22.7)		
	High school	19 (8.4)	31 (13.8)		
	College	18 (8.0)	20 (8.9)		
	Undergraduate or above	6 (2.7)	29 (12.9)		
**Occupational status**				9.934	0.007
	Full-time/part-time	20 (8.9)	29 (12.9)		
	Laid off for illness	6 (2.7)	35 (15.6)		
	Unemployed/retired	55 (24.4)	80 (35.6)		
**Marital status**				0.766	0.381
	Married	73 (32.4)	124 (55.1)		
	Others	8 (3.6)	20 (8.9)		
**Family income** **(per capita monthly, Yuan)**				3.050	0.550
	≤1000	4 (1.8)	10 (4.4)		
	1001–3000	16 (7.1)	30 (13.3)		
	3001–5000	27 (12.0)	37 (16.4)		
	5001–7000	12 (5.3)	32 (14.2)		
	>7000	22 (9.8)	35 (15.6)		
**Personal income** **(monthly, Yuan)**				2.042	0.728
	≤1000	27 (12.0)	41 (18.2)		
	1001–3000	19 (8.4)	46 (20.4)		
	3001–5000	19 (8.4)	32 (14.2)		
	5001–7000	8 (3.6)	14 (6.2)		
	>7000	8 (3.6)	11 (4.9)		
**Diagnosis time**				1.047	0.306
	≤6 months	63 (28.0)	103 (45.8)		
	>6 months	18 (8.0)	41 (18.2)		
**Chemotherapy times**				0.132	0.936
	1–2	28 (12.4)	53 (23.6)		
	3–4	32 (14.2)	56 (24.9)		
	≥5	21 (9.3)	35 (15.6)		
**Surgical approach**				3.560	0.169
	simple mastectomy	64 (28.4)	97 (43.1)		
	local mastectomy or reconstruction	17 (7.6)	47 (20.9)		
**Pathological stage**				2.211	0.331
	Stage Ⅰ	18 (8.0)	40 (17.8)		
	Stage Ⅱ	44 (19.6)	81 (36.0)		
	Stage Ⅲ	19 (8.4)	13 (5.8)		
**Comorbidities**				4.951	0.084
	0	52 (23.1)	92 (40.9)		
	1–2	19 (8.4)	45 (20.0)		
	≥3	10 (4.4)	7 (3.1)		

### Comparison of FCR dimension scores in patients with or without clinically relevant FCR

The total FCRI score among patients with breast cancer undergoing chemotherapy was 43.19 ± 22.83. Among the dimensions of FCRI, the coping strategy dimension had the highest score (15.64 ± 10.05), while the insight dimension had the lowest score (2.17 ± 2.44). Because the total score of the FCRI scale does not have a defined cutoff value for clinical significance, we used a score of FCRI-SF ≥13 as an indicator of FCR with clinical significance based on the cutoff value of the FCRI severity subscale (FCRI-SF) [26, 27]. The results revealed that 64% (n = 144) of patients with breast cancer undergoing postoperative chemotherapy in this study experienced clinically relevant FCR, whereas 36% (n = 81) did not. Further details regarding the differences in scores for each dimension between the groups with and without clinically relevant FCR are presented in Table 2.

**Table 2:** Difference in FCR dimension scores for participants with or without clinically relevant FCR (N = 225).

FCR Dimensions	Dimension score *M(P*_*25*,_*P*_*75*_*)*		Mann–Whitney U	
	FCRI-SF<13	FCRI-SF≧13	Z	*P*
**Total score**	17.0 (7.5, 38.0)	55.0 (43.3, 65.0)	–9.59	<0.001
**Trigger**	4.0 (1.0, 9.0)	13.0 (10.0, 16.0)	–8.665	<0.001
**Severity**	8.0 (6.0, 10.0)	16.0 (14.0, 18.0)	–12.467	<0.001
**Psychological distress**	0.0 (0.0, 2.0)	5.0 (3.0, 8.0)	–9.052	<0.001
**Functional impairments**	0.0 (0.0, 4.0)	6.0 (3.0, 11.0)	–6.865	<0.001
**Insight**	0.0 (0.0, 1.0)	3.0 (1.0, 5.0)	–7.825	<0.001
**Reassurance**	0.0 (0.0, 4.0)	4.0 (1.0, 6.0)	–4.383	<0.001
**Coping strategies**	5.0 (0.0, 16.5)	20.5 (16.0, 25.0)	–6.683	<0.001

### Relationship between symptom experience and FCR

**Fig 1:**
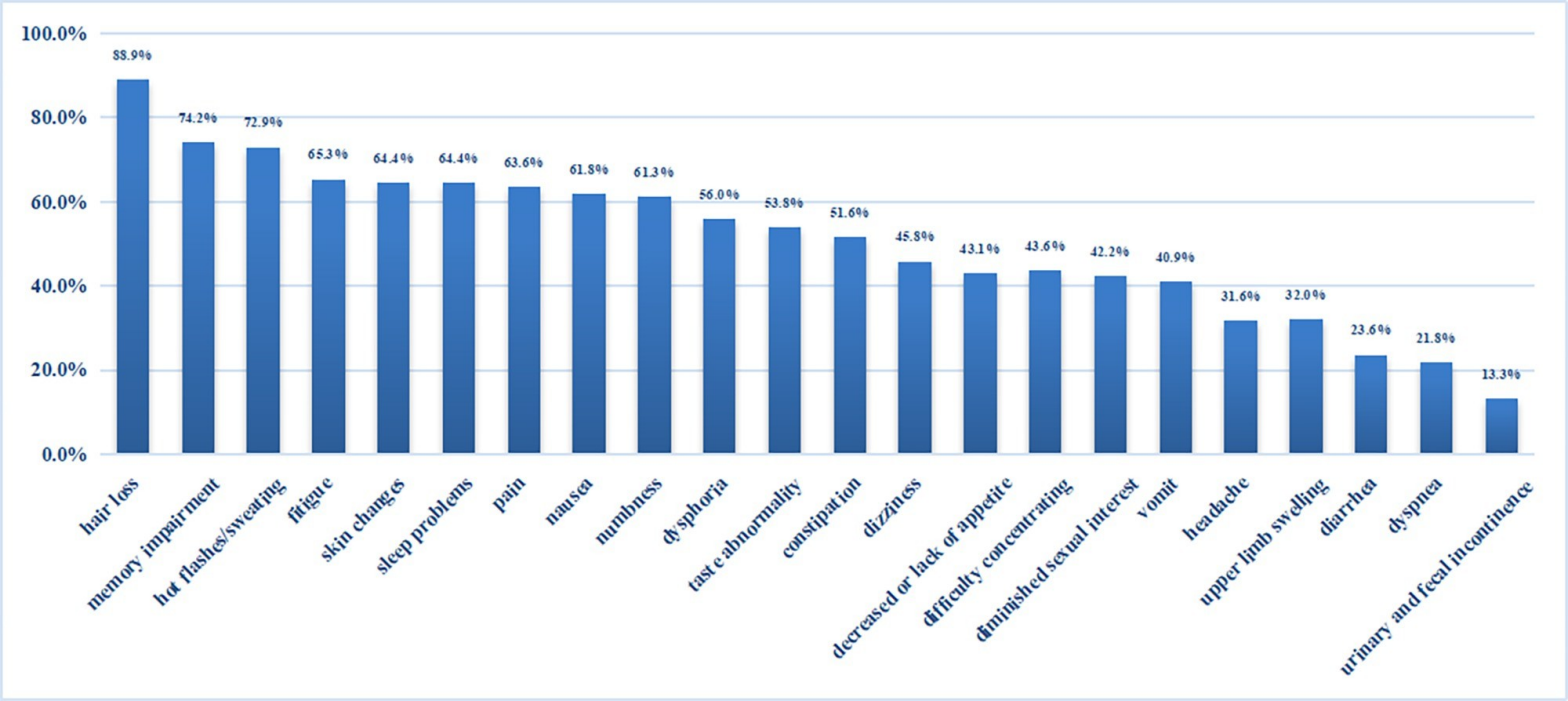
Incidence of single symptoms.

This study assessed 23 symptoms experienced by patients with breast cancer undergoing chemotherapy, with nine having an incidence of 60% or higher (Fig 1).

The total score of the SEI was 27.41 ± 16.77, consisting of symptom severity (16.91 ± 8.70) and symptom distress (10.50 ± 8.89) dimension scores. The Mann–Whitney U test indicated significant differences in total symptom experience and most specific symptoms between patients with or without clinically relevant FCR but no differences in some individual symptoms (Table 3).

**Table 3:** Difference in scores of symptom experience for participants with or without clinically relevant FCR (N = 225).

Symptom experience	*M(P*_*25*_, *P*_*75*_*)*		Mann–Whitney U	
	FCRI-SF<13	FCRI-SF≧13	Z	*P*
**hair loss**	4.0 (2.0, 5.0)	3.0 (2.0, 5.0)	–0.537	0.591
**hot flashes/sweating**	1.0 (0.5, 2.0)	1.0 (0.0, 3.0)	–1.314	0.189
**sleep problems** *	1.0 (0.0, 2.0)	2.0 (0.0, 3.0)	–2.232	0.026
**nausea**	1.0 (0.0, 2.0)	2.0 (0.0, 3.0)	–1.609	0.108
**memory impairment** *	1.0 (0.0, 2.0)	2.0 (1.0, 2.0)	–3.382	0.001
**skin changes** *	1.0 (0.0, 2.0)	2.0 (0.0, 2.0)	–2.836	0.005
**fatigue**	1.0 (0.0, 2.0)	1.0 (0.0, 2.0)	–1.940	0.052
**pain** *	1.0 (0.0, 2.0)	2.0 (0.0, 2.0)	–2.729	0.006
**numbness** *	1.0 (0.0, 2.0)	1.0 (0.0, 2.0)	–2.157	0.031
**dysphoria** *	0.0 (0.0, 1.5)	1.5 (0.0, 2.0)	–4.583	<0.001
**taste abnormality**	0.0 (0.0, 2.0)	1.0 (0.0, 2.0)	–0.949	0.343
**constipation** *	0.0 (0.0, 2.0)	1.0 (0.0, 2.0)	–2.101	0.036
**dizziness**	0.0 (0.0, 1.0)	0.0 (0.0, 2.0)	–1.919	0.055
**decreased or lack of appetite**	0.0 (0.0, 1.0)	0.0 (0.0, 2.0)	–1.537	0.124
**difficulty concentrating** *	0.0 (0.0, 1.0)	1.0 (0.0, 1.0)	–3.238	<0.001
**diminished sexual interest** *	0.0 (0.0, 1.0)	1.0 (0.0, 2.0)	–2.832	0.005
**vomiting** *	0.0 (0.0, 2.0)	0.0 (0.0, 2.0)	–2.164	0.030
**headache** *	0.0 (0.0, 0.0)	0.0 (0.0, 2.0)	–2.309	0.021
**upper limb swelling** *	0.0 (0.0, 0.0)	0.0 (0.0, 1.8)	–3.315	0.001
**diarrhea** *	0.0 (0.0, 0.0)	0.0 (0.0, 1.0)	–2.437	0.015
**dyspnea**	0.0 (0.0, 0.0)	0.0 (0.0, 0.0)	–0.048	0.961
**urinary incontinence**	0.0 (0.0, 0.0)	0.0 (0.0, 0.0)	–1.012	0.311
**fecal incontinence**	0.0 (0.0, 0.0)	0.0 (0.0, 0.0)	–0.176	0.860
**dimension of symptom severity** *	14.0 (9.0, 19.0)	17.0 (12.0, 22.8)	–3.245	0.001
**dimension of symptom distress** *	6.0 (1.0, 11.0)	11.0 (5.0, 17.0)	–4.185	<0.001
**total symptom experience** *	20 (12.5, 27)	28.0 (18.0, 40.8)	–3.911	<0.001

*represents a statistical difference between the groups with or without clinically significant FCR.

**Fig 2:**
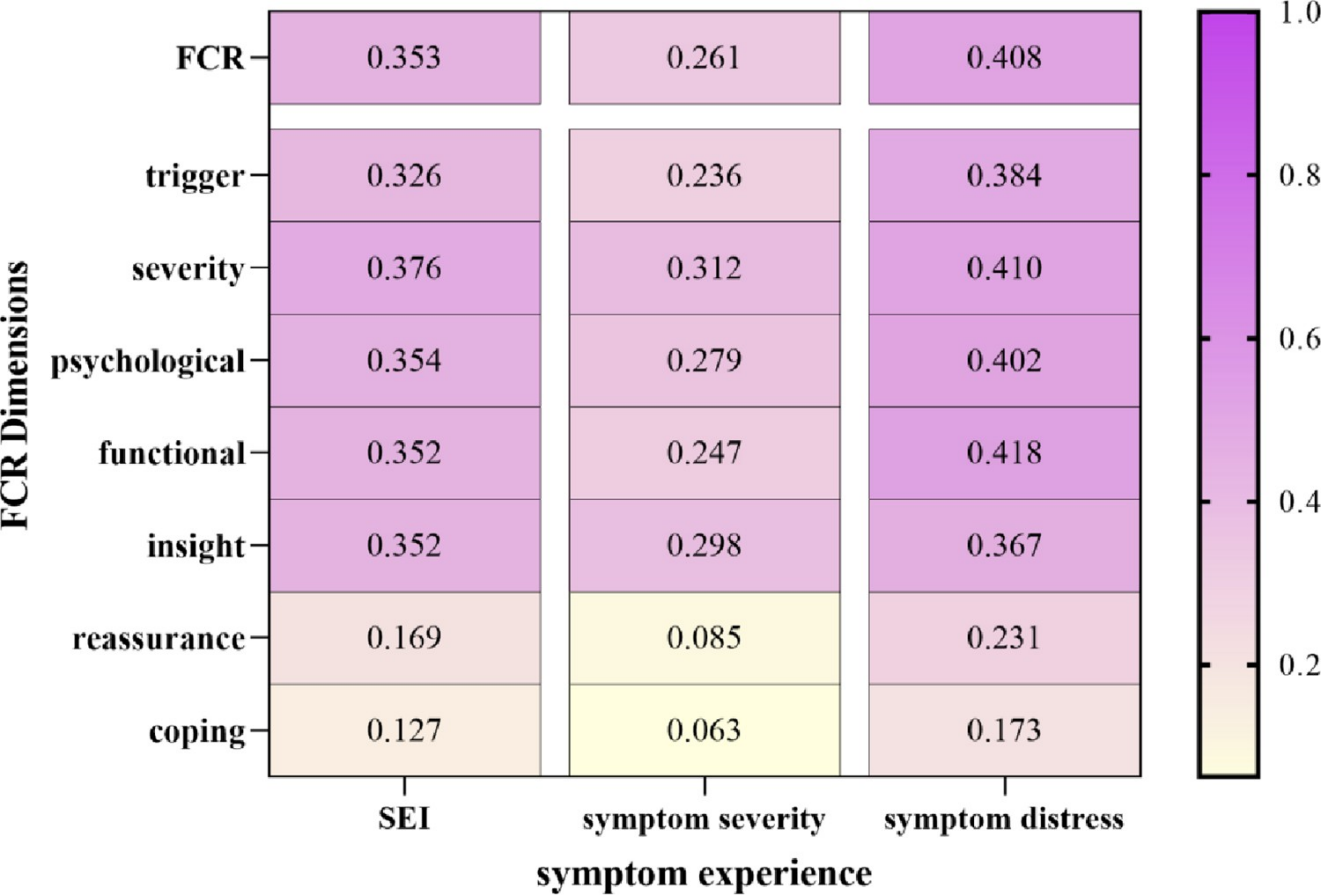
Correlation between symptom experience and FCR.

Spearman’s correlation analysis revealed a significant positive correlation between symptom experience and FCR (r = 0.353, P < 0.001), as shown in Fig 2.

Among the dimensions of FCR, symptom distress showed the strongest correlation with the functional impairment dimension (r = 0.418, P < 0.001), whereas symptom experience demonstrated the weakest correlation with the reassurance dimension (r = 0.196, P < 0.011). No significant correlations were observed between symptom severity and reassurance or coping strategy dimensions. Additionally, symptom experience was not correlated with coping strategies in FCR.

Multiple linear regression analysis was conducted using FCR. Taking the total FCRI score as the dependent variable and variables that had a statistical relationship with FCR in the analysis of variance or T-test (S1 Table) or variables that have clinical significance to FCR as independent variables (age, education level, surgical approach, occupational status, and total symptom experience score), multiple linear regression was performed. As a result, symptom experience, age, and education level entered the regression equation with *F* = 28.447, *R*^*2*^ = 0.279, adjusted *R*^*2*^ = 0.270, *P* < 0.001, accounting for 27.0% of the FCR variance. The results indicated that symptom experience (b = 0.511, t = 6.474, P < 0.001), age (b = –0.591, t = –4.201, P < 0.001), and educational level (b = 4.147, t = 3.955, P < 0.001) had a statistically significant correlation with FCR. Higher scores for symptom experience, younger age, and higher educational level were associated with higher FCR scores. Among these variables, symptom experience had the strongest correlation with FCR, with a beta value of 0.371 (Table 4).

**Table 4:** Multivariate analysis of FCR (step by step).

Constant	*B*	*SE*	*Beta*	*t*	*P*	*B for 95% CI*	
						Lower limit	Upper limit
	46.642	8.518		5.475	<0.001	29.854	63.430
**symptom experience**	0.511	0.079	0.371	6.474	<0.001	0.355	0.667
**age**	–0.591	0.141	–0.246	–4.201	<0.001	–0.868	–0.314
**education level**	4.147	1.049	0.232	3.955	<0.001	2.080	6.214

*F* = 28.447, *R*^*2*^ = 0.279, adjusted *R*^*2*^ = 0.270, *P*<0.001, *95% CI* represents 95% confidence interval

## Discussion

Current studies have consistently shown that FCR among patients with breast cancer is very common and cannot be ignored, impacting overall quality of life and social functioning, with a clear relationship between the occurrence and severity of physical or psychological symptoms and FCR [14, 15, 17–19]. However, the association between symptom experience (the total perception of various symptom dimensions) and FCR remains unclear. The results of this study revealed that 64% of patients with breast cancer undergoing postoperative chemotherapy experienced clinically relevant FCR. Furthermore, a significant positive correlation between symptom experience and FCR was observed, and patients with or without clinically relevant FCR exhibited differences in their symptom experiences, including some specific symptoms.

The proportion of participants with clinically relevant FCR (64%) in this study was similar to the findings of Chinese researchers, including Ren (61.0%) [11], Niu (68.4%) [12], Zhang (69.5%) [30], and other international scholars (60%) [31], indicating that the majority of postoperative patients with breast cancer undergoing chemotherapy experience clinical relevance. Although the average score of the severity subscale was consistent with other research results, the total FCRI score in this study was lower than that reported by researchers who included patients using the same scale for various cancer types [25, 32–35]. There are several possible explanations for these results. First, the disease type of the participants in this study was specifically breast cancer, excluding other types of tumors such as lung cancer or melanoma, which have been reported to have the highest FCR [31]. Breast cancer has a relatively higher survival rate compared to other cancers [36]. Furthermore, individuals with a history of recurrence tended to experience higher FCR [14]. The participants in this study were patients with stage I to III breast cancer without recurrence or metastasis who were undergoing postoperative chemotherapy. They had higher treatment expectations and better outcomes, which may have contributed to their lower average FCR scores. In addition, it is important to consider the influence of traditional Chinese culture. Chinese people tend to avoid mentioning bad news such as recurrence, metastasis, or death. They tell themselves not to think about whether there will be recurrence or metastasis but to believe in and cooperate with the current treatment. As for the outcome, they believe that it is only necessary to “do everything in one’s power and listen to fate.” Research has shown that breast cancer patients in China have low levels of self-disclosure [37]. Thus, to some extent, participants may hide their true feelings during the survey process, leading to biased results. Another possible reason might be that participants, concerned that survey results could influence nurse-patient relationships, tend to report more favorable outcomes in the presence of researchers and nurses. Nonetheless, the results of this study highlight that more than half of patients with breast cancer experience clinically relevant FCR, indicating the need for ongoing attention from healthcare professionals.

A total of 23 different symptoms were investigated during chemotherapy, and the overall symptom experience score was generally consistent with the results reported by Li et al. in Chinese patients with cancer [29]. Hair loss was the most common symptom (88.9%), followed by memory impairment, hot flashes or sweating, fatigue, skin changes, sleep disorders, pain, nausea, numbness, dysphoria, taste abnormalities, and constipation. These findings are consistent with those of previous research, which also highlighted the prevalence of sleep difficulties, fatigue, pain, anxiety, depression, worry, sadness, nervousness, lack of appetite, and nausea during cancer treatment [38, 39]. These symptoms may be related to the side effects of chemotherapeutic drugs such as docetaxel, which can persist throughout the course of chemotherapy [40]. Vomiting, nausea, sleep problems, constipation, headache, and alopecia were the most distressing symptoms. Although the frequency and severity of symptoms such as dyspnea and diarrhea were not high, they still caused significant disruptions to patients. This suggests the importance of focusing on symptoms that significantly affect patients and providing timely interventions to improve their symptom experience.

Statistically significant positive associations were found between symptom experience and FCR. FCR tended to increase with increase in the severity and distress of symptoms. Previous studies have shown that patients with significant FCR have poorer sleep quality, increased anxiety and depression symptoms, and lower overall quality of life [41, 42]. Patients with higher FCR may also be more vigilant about their symptoms and more likely to report discomfort. Conversely, some studies have consistently shown that patients with more severe symptoms, such as cancer-related fatigue, pain, and sleep disorders, tend to report higher FCR, some of which consider persistent symptoms to be predictors of FCR [4, 17, 43–45]. For example, in this study, symptoms with established psychological components (e.g., difficulty concentrating, diminished sexual interest, memory impairment, fatigue) had a stronger correlation with FCR compared to those where this is less the case (e.g., hot flashes/sweating, diarrhea, nausea, constipation) (S2 Table). Moreover, higher scores for total symptom experience, younger age, and higher education level were significantly associated with FCR in patients with breast cancer undergoing chemotherapy, with symptom experience showing the strongest correlation.

Furthermore, our study confirmed that younger patients tended to experience more clinically relevant FCR, which is consistent with the results of previous studies [22, 46, 47]. The prevalence of breast cancer in younger age groups is increasing in China [3]. These patients face unique challenges related to their families, employment, and the needs of their children. Additionally, the long-term recurrence rate increases with the prolongation of survival in breast cancer [48], and young patients may be more likely to worry about FCR.

We also found that patients with a higher educational level demonstrated a greater tendency toward FCR, consistent with the findings of Mahendran R and Hu Z. [47, 49]. However, it is worth noting that our findings differ from most existing studies. For example, previous reports highlighted a negative correlation between educational level and FCR [50, 51]. These studies suggest that patients with higher education levels can obtain more disease-related information and respond effectively to disease and treatment-related side effects, resulting in lower FCR levels. In contrast, patients with high school or higher levels of education made up a relatively high proportion of the group with clinically significant FCR in this study, and they often had stable careers or jobs, clearer career plans, and higher career pursuits. Interrupting career development due to disease-related obstacles may add a further psychological burden. Different regions, national conditions, and cultural backgrounds may also explain these conflicting conclusions. Further research is required to verify and clarify these contradictory findings in future studies.

### Research limitations

This study has several limitations, the most important of which is its cross-sectional design, which precludes causal inferences. In addition, owing to time and resource constraints, patient data during the rehabilitation period were not collected. This prevented us from comprehensively understanding the dynamic changes in symptom experience and FCR in postoperative patients with breast cancer undergoing chemotherapy. Furthermore, the study only included patients from a single inpatient ward and did not include patients from other settings, such as the day chemotherapy ward or other cancer centers. This may have resulted in some information regarding patients undergoing chemotherapy being overlooked. Notably, the patients included in this study were all undergoing postoperative chemotherapy without recurrence or metastasis, resulting in a highly homogeneous sample. Therefore, we believe that these findings are representative.

## Conclusions

The findings of this study indicate that more than half of the patients with breast cancer undergoing chemotherapy experience clinically relevant FCR. A statistically significant correlation was observed between symptom experience and FCR. The more severe the symptom experienced, the higher the FCR tended to be. Higher scores of total symptom experience, younger age, and higher education level were significantly associated with FCR in patients with breast cancer undergoing chemotherapy, with symptom experience showing the strongest correlation. The relationships between the independent variables and conclusions could not be explored in this study due to its design; thus, further prospective research is needed to better understand the relationship between symptom experience and FCR.

## Supporting information

S1 Table Influences of general information on FCR. (DOCX)

S2 Table Correlation between single symptom experience and FCR. (DOCX)
