# Supplementary material for: Correlation between symptom experience and fear of cancer recurrence in postoperative breast cancer patients undergoing chemotherapy in China: A cross-sectional study
Source: PLoS One. 2024 Sep 18;19(9):e0308907. doi: 10.1371/journal.pone.0308907 (PMC11410183; doi:10.1371/journal.pone.0308907)
Supplement: S1 Table — (DOCX) [file pone.0308907.s001.docx]

## Supporting information

| S1 Table. Influences of general information on FCR （N=225） | | | | |
| --- | --- | --- | --- | --- |
| **Variable** |  | **FCRI**  **（$\bar{X}\pm SD$）** | ***F/t*** | ***P*** |
| **Age, year*** |  |  | 4.786 | ＜0.001 |
|  | ≤30 | 54.88±12.94 |  |  |
|  | 31~40 | 53.17±20.32 |  |  |
|  | 41~50 | 46.06±24.24 |  |  |
|  | 51~60 | 44.23±24.62 |  |  |
|  | ＞60 | 31.11±23.31 |  |  |
| **Educational level*** |  |  | 6.793 | ＜0.001 |
|  | Elementary school or below | 27.37±21.07 |  |  |
|  | Junior high school | 42.51±23.27 |  |  |
|  | High school | 41.26±21.97 |  |  |
|  | College | 46.95±21.03 |  |  |
|  | Undergraduate or above | 55.51±19.00 |  |  |
| **Occupational status*** |  |  | 6.647 | 0.002 |
|  | Full-time/part-time | 46.10±22.72 |  |  |
|  | Laid off for illness | 53.02±21.67 |  |  |
|  | Unemployed/retired | 39.14±22.29 |  |  |
| **Marital status** |  |  | -1.767 | 0.079 |
|  | Married | 42.18±22.90 |  |  |
|  | Others | 50.29±21.34 |  |  |
| **Family income**  **(capita monthly, Yuan)** |  |  | 1.468 | 0.213 |
|  | ≤1000 | 44.21±26.07 |  |  |
|  | 1001~3000 | 42.89±21.08 |  |  |
|  | 3001~5000 | 37.86±22.57 |  |  |
|  | 5001~7000 | 47.39±23.30 |  |  |
|  | ＞7000 | 45.91±22.93 |  |  |
| **Personal income**  **（monthly, Yuan）** |  |  | 1.098 | 0.358 |
|  | ≤1000 | 38.78±24.91 |  |  |
|  | 1001~3000 | 44.02±20.03 |  |  |
|  | 3001~5000 | 45.73±22.91 |  |  |
|  | 5001~7000 | 43.68±25.20 |  |  |
|  | ＞7000 | 48.74±20.54 |  |  |
| **Diagnosis time** |  |  | 0.464 | 0.643 |
|  | ≤6 months | 43.61±22.88 |  |  |
|  | ＞6 months | 42.00±22.82 |  |  |
| **Chemotherapy times** |  |  | 0.728 | 0.484 |
|  | 1~2 | 45.35±22.76 |  |  |
|  | 3~4 | 42.84±22.12 |  |  |
|  | ≥5 | 40.61±24.10 |  |  |
| **Surgical approach*** |  |  | -2.841 | 0.003 |
|  | simple mastectomy | 40.50±23.59 |  |  |
|  | local mastectomy or reconstruction | 49.94±19.35 |  |  |
| **Pathological stage** |  |  | 2.455 | 0.088 |
|  | stage Ⅰ | 48.84±21.07 |  |  |
|  | stageⅡ | 41.44±23.44 |  |  |
|  | stageⅢ | 40.57±22.52 |  |  |
| **Comorbidities numbers** |  |  | 1.384 | 0.253 |
|  | 0 | 44.19±23.10 |  |  |
|  | 1~2 | 43.23±21.59 |  |  |
|  | ≥3 | 34.47±24.49 |  |  |
| ***** represents variables with statistical differences. Statistical analysis: Analysis of variance and t-tests. | | | | |
