# Supplementary material for: Correlation between symptom experience and fear of cancer recurrence in postoperative breast cancer patients undergoing chemotherapy in China: A cross-sectional study
Source: PLoS One. 2024 Sep 18;19(9):e0308907. doi: 10.1371/journal.pone.0308907 (PMC11410183; doi:10.1371/journal.pone.0308907)
Supplement: S2 Table — (DOCX) [file pone.0308907.s002.docx]

## Supporting information

| **S2 Table. Correlation between single symptom experience and FCR（N=225）** | | | |
| --- | --- | --- | --- |
| **Symptom experience** | **FCRI** | | |
|  | ***r*** | ***P*** | |
| **difficulty concentrating*** | 0.410 | | ＜0.001 |
| **dysphoria*** | 0.394 | | ＜0.001 |
| **diminished sexual interest*** | 0.310 | | ＜0.001 |
| **memory impairment*** | 0.281 | | ＜0.001 |
| **fatigue*** | 0.272 | | ＜0.001 |
| **skin changes*** | 0.270 | | ＜0.001 |
| **headache*** | 0.244 | | ＜0.001 |
| **pain*** | 0.231 | | ＜0.001 |
| **sleep problems*** | 0.212 | | 0.001 |
| **dizziness*** | 0.202 | | 0.002 |
| **upper limb swelling*** | 0.196 | | 0.003 |
| **numbness*** | 0.178 | | 0.008 |
| **vomiting*** | 0.177 | | 0.008 |
| **decreased or lack of appetite*** | 0.172 | | 0.010 |
| **constipation*** | 0.162 | | 0.015 |
| **nausea*** | 1.54 | | 0.021 |
| **diarrhea*** | 0.137 | | 0.040 |
| **hot flashes/sweating*** | 0.134 | | 0.045 |
| **taste abnormality** | 0.122 | | 0.067 |
| **dyspnea** | 0.087 | | 0.192 |
| **fecal incontinence** | 0.049 | | 0.463 |
| **urinary incontinence** | 0.044 | | 0.510 |
| **hair loss** | -0.012 | | 0.867 |
| *represents a statistical correlation between single symptom experience and FCR.Statistical analysis: Spearman's correlation. | | | |
